# IRIS manifesting as chronic inflammatory demyelinating polyneuropathy in HIV

**DOI:** 10.1186/1471-2334-14-S3-P72

**Published:** 2014-05-27

**Authors:** Aravind Reghukumar, Athul Gurudas, VS Kiran Kumar, Ranjani Ravi

**Affiliations:** 1 Department of Infectious Diseases, Medical College Hospital, Thiruvananthapuram, India; 2 Cosmopolitan Hospital, Thiruvananthapuram, India

## Background

CIDP manifesting as part of immune response inflammatory syndrome (IRIS) in HIV has not been reported till date.

## Case report

45 year old female was diagnosed to have HIV with a CD4 count of 86. At the time of diagnosis, she was very much emaciated with a BMI of 16, had oral candidiasis and sensory neuropathy with numbness upto both ankles. After ruling out other opportunistic infections, she was started on tenofovir, lamivudine and nevirapine. Three months later, she presented to us with difficulty in walking and dribbling of saliva. History was suggestive of gradual worsening of peripheral neuropathy and progressive ascending limb weakness after initiation of anti retroviral therapy. Bowel and bladder involvement were not present. Clinical examination revealed areflexic quadriparesis with bilateral LMN facial palsy. Evidence of both small and large fiber neuropathy was present in both upper and lower limbs. Digital clawing was noted in both upper limbs in both ulnar and median nerve distribution.CSF study was suggestive of lymphocytic pleocytosis with mild protein elevation. MRI brain and spine were normal. Electromyographic nerve Sconduction studies were consistent with a primary demyelinating polyneuropathy involving all nerves in upper and lower limbs. CSF PCR for Cytomegalovirus, Ebstein barr virus, herpes and mycobacterium tuberculosis were negative. Since duration of symptoms was more than 2 months, a diagnosis of CIDP was made. She was given inj methylprednisolone 1 gm for 5 days followed by tapering doses of prednisolone. There was dramatic improvement in muscle power after initiation of steroids and after 2 weeks she was able to walk without support, facial palsy and digital clawing had recovered.

## Conclusion

The probable explanation is that CIDP in this case occurred as IRIS as evidenced by the improved BMI (19) and CD4 count (170) at the time of presentation.

